# Factors associated with daily opioid use among aged home care clients: a cross-sectional analysis of Resident Assessment Instrument data

**DOI:** 10.1007/s41999-021-00533-0

**Published:** 2021-07-09

**Authors:** Heidi Mörttinen-Vallius, Sirpa Hartikainen, Heini Huhtala, Lauri Seinelä, Esa Jämsen

**Affiliations:** 1 Faculty of Medicine and Health Technology, Tampere University, N33014 Tampere, Finland; 2 Research Centre of Geriatric Care, School of Pharmacy, University of Eastern Finland, Kuopio, Finland; 3 Faculty of Social Sciences, Tampere University, N33014 Tampere, Finland; 4 Faculty of Medicine and Health Technology and Gerontology Research Centre GEREC, Tampere University, FIN-33014 Tampere, Finland; 5 Tampere University Hospital, P.O. Box 2000, FIN-33521 Tampere, Finland

**Keywords:** Aged, Cognitive impairment, Home care, Opioids, Resident Assessment Instrument

## Abstract

**Purpose:**

To examine which client characteristics and other factors, including possible adverse effects, identified in the Resident Assessment Instrument—Home Care (RAI-HC) are associated with daily opioid use among aged home care clients.

**Methods:**

The study sample comprised 2584 home care clients aged ≥ 65 years, of which 282 persons used opioids daily. Clients using opioids less than once daily were excluded. The cross-sectional data were gathered from each client’s first assessment with the RAI-HC during 2014. Multivariable logistic regression was used to study associations of daily opioid use with the clients’ characteristics and symptoms.

**Results:**

Cognitive impairment was associated with less frequent opioid use after adjusting for pain-related diseases, disabilities and depressive symptoms (OR 0.43, 95% CI 0.32–0.58). The association was not explained by the estimated severity of pain. Osteoporosis, cancer within previous 5 years and greater disabilities in Instrumental Activities of Daily Living (IADL) were associated with daily opioid use regardless of the estimated severity of pain. Depressive symptoms and Parkinson’s disease were associated with daily opioid use only among clients with cognitive impairment, and disabilities in Activities of Daily Living, cancer, arthritis, fractures and pressure ulcers only among clients without cognitive impairment. Constipation was the only adverse effect associated with daily opioid use.

**Conclusion:**

The pain of home care clients with cognitive impairment may not be treated optimally, whereas there might be prolonged opioid use without a sufficient evaluation of current pain among clients with osteoporosis, cancer within previous 5 years and disabilities in IADLs.

## Key summary points

**Aim:**

To examine which client characteristics and other factors, including possible adverse effects, identified in the Resident Assessment Instrument-Home Care (RAI-HC) are associated with daily opioid use among aged home care clients.

**Findings:**

After adjustment for pain-related diseases, disabilities, depressive symptoms and the estimated severity of pain, daily opioid use was associated with osteoporosis, cancer within previous 5 years and greater disabilities in Instrumental Activities of Daily Living, and cognitive impairment was associated with less frequent opioid use. Constipation was the only adverse effect identified in the RAI-HC associated with daily opioid use.

**Message:**

The pain of home care clients with cognitive impairment may not be treated optimally, whereas there might be prolonged opioid use without a sufficient evaluation of current pain among clients with osteoporosis, cancer within previous 5 years and disabilities in Instrumental Activities of Daily Living.

## Introduction

Around one in ten home-dwelling older adults uses opioids, and the proportion is even greater when shorter periods of use are counted [1–4]. The most common indications for long-term opioid use among home care clients are musculoskeletal disorders, mainly vertebral osteoporotic fractures, degenerative spinal disorders and osteoarthritis [1]. Opioid use has been associated with the female gender, lower education and socioeconomic position, and polypharmacy among older community-dwelling adults [2, 3, 5], but associations with age, cardiovascular and pulmonary diseases, diabetes and impairment in activities of daily living have varied between studies [2, 3, 5–7]. There has been concerns that older adults with cognitive impairment are at increased risk for undertreated pain [7–9], and the prevalence of pain-related diseases has not explained the difference [4, 10, 11]. As far as we know, factors associated with opioid use in home care clients with cognitive impairment have not been studied.

The number needed to harm of opioid use is smaller among older adults (≥ 65 years) compared to younger persons [12], but the evidence for the adverse effects and events of long-term opioid use is insufficient. Discontinuation of opioid treatment is common due to adverse effects or insufficient pain relief [13, 14], but some adverse effects tend to diminish over time [12, 14]. Presumably long-term opioid users tolerate opioids, and their adverse effects may, therefore, differ from the populations in randomised controlled trials of short-term use.

The aim of this study was to examine which client characteristics and other factors, including possible adverse effects, identified in the Resident Assessment Instrument—Home Care (RAI-HC) are associated with daily opioid use among aged home care clients. In addition, it was investigated whether factors associated with opioid use differ between clients with and without cognitive impairment.

## Methods

This study was based on cross-sectional data from the RAI-HC. The study population consisted of all persons aged 65 years or older receiving regular home care services at least once per week in the area of Tampere city (population circa 222,000, of which 3,535 were registered as home care clients), Finland, who were assessed at least once with the RAI-HC during 2014. Home care clients having only an incomplete RAI-HC assessment or whose first assessment created during 2014 were not finished until 2015 were excluded.

**Fig. 1:**
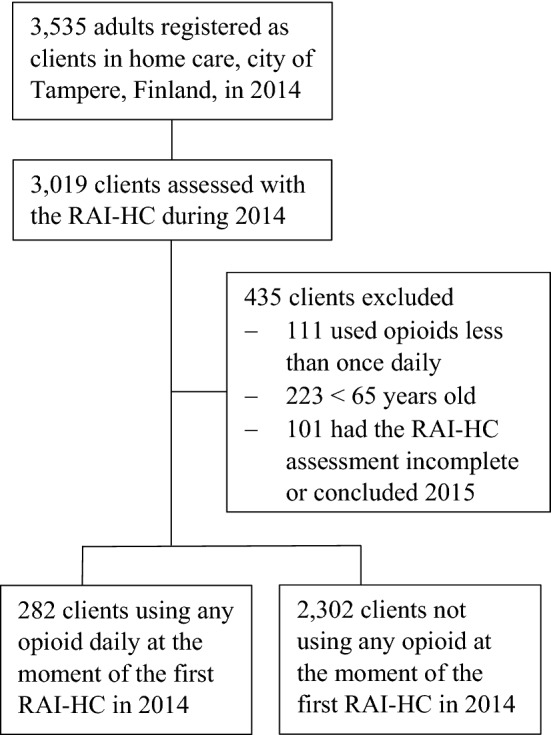
Sample selection

Home care clients using any opioid daily (282 persons) were identified using the medication list recorded in each client’s RAI-HC. Opioid use was checked manually by one author (HMV) against the electronic medical records covering the home care, primary health care and secondary care hospital in the area. Clients who used opioids as needed (i.e., less frequently than once daily) were excluded because their actual opioid use at the time of the assessment could not be verified retrospectively. The sample selection is described in Fig. 1.

### Measures

The data were gathered from the clients’ first RAI-HC assessment during 2014. The RAI-HC is an internationally used tool for the comprehensive, multidimensional assessment of older people receiving supportive services in community-based settings (www.interrai.org), and its reliability and validity have been reported in previous studies [15, 16]. Tampere city has utilised the RAI-HC in the development of home care services since 2007 and all clients were assessed by a member of the educated nursing staff. Data on clients’ socio-demographic and health status, disabilities, comorbidities, use of psychotropic medication, and symptoms that could represent the common adverse effects of opioids were gathered from the RAI-HC.

The RAI-HC provides several standardised sum scales describing clients’ disabilities and health status, which were used in this study. The Pain scale [17] assesses pain frequency (from no pain to pain several times per day) and pain intensity (from no pain to severe pain) over the seven days before the assessment. It ranges from 0 to 3, and a score of 0 refers to no pain and 3 refers to severe daily pain. Disabilities in daily activities were measured using the Activities of Daily Living (ADL_28) [18] and the Instrumental Activities of Daily Living (IADL) [15] scales. On both scales, higher scores indicate greater dependence, and the ADL was categorized as having disability when scores were ≥ 1. The CHESS (Changes in Health, End-Stage Disease, Signs and Symptoms Scale) [19] measures the stability of health status, with scores ≥ 3 indicating moderate to very high health instability. The MAPLe (Method for Assigning Priority Levels) [20] predicts the need for long-term care, and scores of 4–5 refer to high or very high risk. The Depression Rating Scale (DRS) [21] scores ≥ 3 indicate the possibility of depression. In this study persons with the Cognitive Performance Scale (CPS) [22] scores ≥ 2 were considered to have cognitive impairment, as these scores indicate at least mild cognitive impairment. The CPS was chosen instead of dementia diagnoses, as it was assumed that some clients have cognitive impairment without specific diagnosis, e.g. due to refusal of diagnostic examinations.

Comorbidities that could affect opioid use [3, 23], could restrict the use of non-steroidal anti-inflammatory drugs [24], or are known indications for opioid use [1], included any documented cancer during the previous 5 years (except basalioma), any diagnosed cognitive or psychiatric disorder, a history of stroke, Parkinson’s disease, cardiac diseases (arrythmias, ischemic heart disease or congestive heart failure), chronic renal failure, peripheral arterial disease or venous insufficiency, diabetes, pressure ulcers, arthritis, osteoporosis or a history of any fracture affecting the present health status. Constipation, vomiting, dizziness, unsteady gait, urinary difficulties and hallucinations were regarded as symptoms that could represent the common adverse effects of opioids in the RAI-HC [12, 14, 25, 26].

The study protocol was approved by the City of Tampere. Neither ethics board approval nor informed patient consent was required by Finnish law, as the retrospective study protocol did not influence the home care clients’ treatment and the clients were not contacted.

### Statistical analysis

SPSS version 25 was used to analyse the data. Descriptive analyses were completed using means with standard deviations (SD) or percentages. Comparisons between opioid users and non-users were made using the independent samples *t* test for continuous variables and cross-tabulation with the Chi-square test for nominal variables.

Logistic regression was used to study the associations of daily opioid use with selected client characteristics, symptoms and the RAI-HC measures. In addition to univariable analysis, several multivariable models were constructed. For the analysis concerning factors associated with opioid use, there were two multivariable models. The first included age, gender, comorbidities that could affect opioid use (a history of stroke, Parkinson’s disease, osteoporosis, arthritis, history of fractures affecting client’s present health status, cancer within previous 5 years, pressure ulcers, cardiac diseases, peripheral arterial disease or venous insufficiency, diabetes and chronic renal failure) and the RAI-HC measures [disability in ADLs (≥ or < 1), performance in IADLs, unstable health state (CHESS ≥ or < 3), depressive symptoms (DRS ≥ or < 3) and cognitive status (CPS ≥ or < 2)]. The MAPLe was not included in the analyses due to collinearity with the CPS and the ADL. The second model included the same variables and the Pain scale, which was added to see if the estimated severity of pain explained the associations founded.

For the analysis concerning the possible adverse effects of opioid use, there were three multivariable models. The first one included age, gender, cognitive status (CPS ≥ or < 2) and depressive symptoms (DRS ≥ or < 3), any psychiatric disorder and the comorbidities mentioned above, except pressure ulcers, which were not considered as confounding factors for the selected symptoms. Then, data about the use of antidepressants, antipsychotics and benzodiazepines and related drugs were added to the second model. The number of medications was not used due to collinearity with separate drugs. Finally, the Pain scale was added to create multivariable model 3.

To estimate the effect of cognitive impairment, the analyses concerning factors associated with daily opioid use were repeated in the subpopulations of clients with and without cognitive impairment.

The results were expressed as odds ratios (ORs) with 95% confidence intervals (CIs). Missing values were not included in the analyses. Missing values considered mostly the same persons, and there were a maximum of 48 persons (of which a maximum were 2 opioid users) missing from the logistic regression analyses. Goodness-of-fit was assessed with the Hosmer–Lemeshow test. Variance inflation factor (VIF) was calculated to measure multicollinearity among the variables in the different models. VIF values over 4, considered indicative of multicollinearity, were not found. Overall model quality was assessed using the Akaike information criterion (AIC).

## Results

The study included altogether 2584 home care clients (mean age 82.7 years, range 65–104 years), of whom 282 used any opioid daily at the time of their first RAI-HC assessment in 2014. Compared to non-users, opioid users were more often women (79% vs 72%) and had less frequently cognitive impairment (37% vs 52%), but more frequently depressive symptoms (24% vs 14%) and disabilities in ADLs or IADLs. Cancer within previous 5 years, Parkinson’s disease, pressure ulcers, osteoporosis, arthritis and a history of fractures were more common among opioid users than non-users. There were more users of antidepressants and benzodiazepines and related drugs but fewer users of antipsychotics among opioid users. The characteristics of daily opioid users and non-users are presented in detail in Table 1.

**Table 1:** Characteristics of home care clients according to their opioid use

Characteristics	Daily opioid users (*n* = 282)		Non-users (n = 2302)		*P* value
	*n*	%	*n*	%	
Age, years [mean (SD)]		82.8 (7.3)		82.7 (7.3)	0.753
Gender, female	224	79.4	1657	72.0	0.008
Education					0.351
Primary education or less	169	67.3	1444	69.3	
Secondary education	73	29.1	535	25.7	
University degree	9	3.6	105	5.0	
Walking aid at home	219	77.7	1190	51.8	< 0.001
Disability in Activities of Daily Living (ADL ≥ 1)	92	32.9	588	25.8	0.012
Performance in Instrumental ADLs					0.012
0–7	71	25.4	778	34.1	
8–14	125	44.6	881	38.7	
15–21	84	30.0	620	27.2	
Cognitive performance scale (CPS) ≥ 2	104	37.1	1180	51.8	< 0.001
Unstable health state (CHESS ≥ 3)	40	14.3	230	10.2	0.035
MAPLe^a^ high to very high (≥ 4)	125	45.0	1264	56.0	< 0.001
Depression rating scale (DRS) ≥ 3	68	24.3	320	14.0	< 0.001
Body mass index [mean (SD)]		26.8 (6.2)		26.5 (5.6)	0.336
*Chronic conditions*					
Cardiac disease^b^	152	54.1	1146	50.0	0.191
Diagnosed dementia, any	74	26.3	952	41.5	< 0.001
Diabetes	85	30.2	672	29.3	0.743
Psychiatric disorder, any	55	19.6	432	18.8	0.762
Osteoporosis	101	35.9	383	16.7	< 0.001
Peripheral arterial disease or venous insufficiency	32	11.4	232	10.1	0.506
History of stroke	18	6.4	209	9.1	0.131
Chronic renal failure	27	9.6	198	8.6	0.585
Arthritis	37	13.2	188	8.2	0.005
Cancer (within previous 5 years)	32	11.4	180	7.8	0.042
History of any fracture affecting the present health status	33	11.7	156	6.8	0.003
Pressure ulcer	24	8.5	87	3.8	< 0.001
Parkinson’s disease	14	5.0	57	2.5	0.016
Number of medications					< 0.001
0–4	2	0.7	232	10.1	
5–8	41	14.5	783	34.2	
9 +	239	84.8	1276	55.7	
Antipsychotic use	28	9.9	356	15.5	0.013
Benzodiazepines and related drugs	143	50.7	853	37.2	< 0.001
Antidepressant use	97	34.4	555	24.2	< 0.001
Presence of neuropsychiatric symptoms^c^	30	10.6	293	12.7	0.314

^a^Method for Assigning Priority Levels; *predictor of admission to residential care*

^b^One or several of the following: cardiac arrhythmias, ischemic heart disease or congestive heart failure

^c^One or several of the following: wandering, verbal or physical aggression, oppositional or socially inappropriate behaviour

### Client characteristics associated with daily opioid use

Daily opioid use was associated with osteoporosis, arthritis, Parkinson’s disease, cancer within previous 5 years, pressure ulcers, depressive symptoms and more severe disabilities in IADLs in the adjusted logistic regression model (Table 2). Cognitive impairment was associated with less frequent opioid use. When adjusted also for the Pain scale, opioid use was still associated with osteoporosis, cancer within previous 5 years, and more disabilities in IADLs. Clients with cognitive impairment were using opioids more seldom regardless of the estimated severity of pain. Daily opioid use was not associated with age, gender, a history of fractures, chronic renal failure, cerebrovascular or cardiovascular diseases, nor with diabetes or an unstable health state in the adjusted models.

**Table 2:** Associations of daily opioid use with sociodemographic and clinical characteristics among aged home care clients

Characteristics	Unadjusted	Multivariable model 1	Multivariable model 2
	OR (95% CI)	Adjusted OR (95% CI)	Adjusted OR (95% CI)
Age (year)	1.00 (0.99–1.02)	0.99 (0.97–1.01)	0.99 (0.97–1.01)
Gender, female	**1.50** (1.11–2.04)	1.20 (0.85–1.68)	0.99 (0.69–1.42)
Disability in ADLs (≥ 1)	**1.41** (1.08–1.84)	1.20 (0.86–1.67)	1.14 (0.80–1.61)
Performance in IADLs			
0–7	1	1	1
8–14	**1.56** (1.14–2.11)	**1.63** (1.17–2.25)	**1.62** (1.15–2.28)
15–21	**1.49** (1.06–2.07)	**1.68** (1.10–2.56)	**1.89** (1.21–2.96)
Unstable health state (CHESS ≥ 3)	**1.47** (1.03–2.11)	1.16 (0.78–1.74)	0.84 (0.55–1.28)
Depressive symptoms (DRS ≥ 3)	**1.97** (1.46–2.65)	**2.05** (1.48–2.85)	1.25 (0.88–1.78)
Cognitive impairment (CPS ≥ 2)	**0.55** (0.43–0.71)	**0.43** (0.32–0.58)	**0.54** (0.40–0.74)
Chronic conditions			
Arthritis	**1.70** (1.17–2.47)	**1.56** (1.05–2.33)	1.35 (0.89–2.07)
Cancer (within previous 5 years)	**1.51** (1.01–2.25)	**1.73** (1.13–2.64)	**1.59** (1.02–2.49)
Cardiac disease	1.18 (0.92–1.51)	1.14 (0.87–1.50)	1.09 (0.82–1.45)
Chronic renal failure	1.13 (0.74–1.72)	1.02 (0.66–1.60)	0.93 (0.58–1.48)
Diabetes	1.05 (0.80–1.37)	1.17 (0.88–1.57)	1.28 (0.94–1.73)
History of any fracture affecting the present health status	**1.82** (1.22–2.71)	1.46 (0.96–2.23)	1.23 (0.79–1.91)
History of stroke	0.68 (0.42–1.12)	0.69 (0.41–1.16)	0.73 (0.42–1.26)
Osteoporosis	**2.80** (2.14–3.66)	**2.60** (1.95–3.49)	**2.29** (1.68–3.12)
Parkinson’s disease	**2.06** (1.13–3.74)	**1.93** (1.01–3.69)	1.98 (0.99–3.97)
Peripheral arterial disease or venous insufficiency	1.14 (0.77–1.69)	0.94 (0.62–1.44)	0.80 (0.52–1.24)
Pressure ulcer	**2.36** (1.47–3.77)	**2.00** (1.20–3.33)	1.40 (0.81–2.43)
Pain scale			
No	1		1
Less than daily	3.89 (2.32–6.52)		3.42 (2.02–5.78)
Daily, mild to moderate	8.17 (5.07–13.17)		6.67 (4.08–10.89)
Daily, severe	25.47 (15.42–42.07)		20.15 (11.87–34.22)

Model 1 adjusted for all factors mentioned above except the Pain scale (Hosmer–Lemeshow p value 0.252). Model 2 adjusted for all factors mentioned above (Hosmer–Lemeshow p-value 0.470)

### Symptoms associated with daily opioid use

Of the symptoms that could represent the adverse effects of opioid use, only constipation was significantly associated with daily opioid use after adjusting for confounding factors (adjusted OR_a_ 2.84, 95% CI 1.08–7.44). Unsteady gait, fear of falling and a lack of appetite were more frequent among opioid users than non-users. However, after adjusting for confounding factors, opioid use was not associated with these symptoms, nor with vomiting, difficulties in urination or hallucinations. The frequency of dizziness was about the same among opioid users and non-users (26.6% vs 27.7%) but after adjustments, dizziness was associated with less frequent opioid use [OR_a_ 0.59 (0.43–0.80)]. Compared to non-users, home care clients using opioids daily considered their health state more frequently poor regardless of age, gender, comorbid diseases, cognitive impairment, depressive symptoms and psychotropic medicines used, but the association was explained by the severity of pain. The frequencies of symptoms and associations with opioid use after adjustments are summarised in Table 3.

**Table 3:** Associations of different symptoms with daily opioid use among aged home care clients

Symptoms	Prevalence of symptoms according to status of opioid use		Univariate	Adjusted models 1	Adjusted models 2	Adjusted models 3
	Users (*n* = 282)	Non-users (*n* = 2302)	OR (95% CI)	OR (95% CI)	OR (95% CI)	OR (95% CI)
	%	%				
Unsteady gait	82.3	69.2	**2.07** (1.50–2.84)	**1.72** (1.23–2.40)	**1.64** (1.17–2.29)	1.19 (0.84–1.69)
Does not go outside because of fear of falling	63.5	47.3	**1.94** (1.50–2.50)	**1.69** (1.28–2.22)	**1.65** (1.25–2.19)	1.23 (0.92–1.64)
Dizziness within the last three days	26.6	27.7	0.94 (0.71–1.25)	0.77 (0.57–1.03)	0.75 (0.55–1.00)	**0.59** (0.43–0.80)
Difficulties in urination in at least two of the last three days	6.4	6.3	1.01 (0.61–1.68)	0.86 (0.51–1.45)	0.84 (0.50–1.44)	0.66 (0.38–1.15)
Constipation in last three days	3.2	0.9	**3.75** (1.69–8.31)	**3.65** (1.55–8.57)	**3.91** (1.66–9.21)	**2.84** (1.08–7.44)
Lack of appetite in at least two of the last three days	12.1	7.7	**1.65** (1.12–2.44)	1.50 (0.99–2.26)	1.42 (0.93–2.15)	1.32 (0.85–2.04)
Vomiting in at least 2 of the last 3 days	0.7	0.7	1.09 (0.25–4.77)	0.73 (0.15–3.44)	0.64 (0.13–3.07)	0.69 (0.13–3.63)
Delusions within the last 3 days	1.8	5.3	**0.32** (0.13–0.79)	**0.30** (0.12–0.77)	**0.34** (0.13–0.88)	0.39 (0.15–1.03)
Hallucinations within the last 3 days	3.2	3.1	1.03 (0.51–2.09)	1.03 (0.48–2.23)	1.03 (0.47–2.25)	1.08 (0.48–2.42)
Poor self-rated health	45.0	26.3	**2.29** (1.78–2.95)	**1.76** (1.33–2.32)	**1.67** (1.26–2.22)	1.02 (0.75–1.38)

Models 1: symptom, age, gender, CPS (≥ or < 2), DRS (≥ or < 3), any psychiatric disorder, a history of stroke, Parkinson’s disease, cancer, cardiac disease, chronic renal failure, peripheral arterial disease or venous insufficiency, diabetes, arthritis, osteoporosis, a history of any fracture affecting the present health status. Models 2: factors in model 1 + use of antipsychotics, antidepressants, and benzodiazepines and related drugs. Models 3: factors in model 1 and 2 + the Pain scale. The Hosmer–Lemeshow test found poor goodness of fit for models 1 urination and vomiting only

### The effect of cognitive impairment

The results from the analyses of the subpopulations of home care clients with and without cognitive impairment are presented in the Appendix. After adjustments, disability in ADLs was associated with daily opioid use among clients without cognitive impairment [Model 1, OR_a_ 1.91 (1.24–2.94)] but not among clients with cognitive impairment. Depressive symptoms were associated with opioid use after adjustments only among clients with cognitive impairment [Model 1, OR_a_ 2.84 (1.81–4.44)]. In the adjusted regression model, the association of opioid use with arthritis, a history of fractures, cancer within previous 5 years, and pressure ulcers appeared only among clients without cognitive impairment, and the association with Parkinson’s disease and cardiac diseases appeared only among clients with cognitive impairment.

## Discussion

In this cross-sectional study based on the RAI-HC data, older home care clients’ daily opioid use was associated with osteoporosis, arthritis, Parkinson’s disease, cancer within previous 5 years, pressure ulcers, depressive symptoms and disabilities in IADLs. The estimated severity of pain did not explain the association with osteoporosis, cancer within previous 5 years, and IADL disability. Cognitive impairment was associated with less frequent opioid use regardless of the estimated severity of pain, pain-related diseases, disabilities in ADLs and IADLs or depressive symptoms. Among clients with cognitive impairment, opioid use was associated with depressive symptoms, Parkinson’s disease and cardiac diseases, whereas among clients without cognitive impairment, it was associated with disabilities in ADLs, cancer within previous 5 years, arthritis, previous fractures and pressure ulcers. The associations with osteoporosis and disabilities in IADLs appeared in both groups. Constipation was the only adverse effect identified in the RAI-HC associated with daily opioid use. Based on a previous study of the same population [1], opioid users in this study are known to represent mainly long-term users (the median duration of opioid use before study entry 357 days), and the most common opioid used was transdermal buprenorphine (62%), whereas strong opioids (fentanyl, morphine or oxycodone) were used by 18%.

The association between cognitive impairment and less frequent opioid use is seen in some previous studies [7–9], but not in all [3, 27]. A reduced ability to verbalise and remember pain experiences might explain the risk for undertreated pain in persons with dementia [28]. However, in this home care population, the association existed regardless of the estimated severity of pain, which is consistent with previous findings in nursing homes [10, 29]. Hence, it is possible that opioid use is avoided in clients with cognitive impairment because of the fear of adverse effects and events, such as dizziness and the increased risk of falls.

It was expected that the estimated severity of pain would explain the associations between opioid use and client characteristics. However, osteoporosis, cancer within previous 5 years and disabilities in IADLs were associated with daily opioid use regardless of the severity of pain. This finding suggests that the threshold to prescribe opioids for patients with a history of certain diagnoses, like cancer, might be lower. Only about half of the current pain among aged cancer patients is cancer-related [30]. Also, a previous study of the same population showed, that even though 11.4% of opioid users had cancer during the previous 5 years, only 6.4% had current cancer and as few as 3.2% used an opioid for cancer-related pain [1]. So, it could be speculated that some of these persons might use opioids for minor non-malignant pain because of their previous cancer diagnosis. Another explanation for these findings could be that opioid use has continued for a long time without a critical review of its need. Following from the cross-sectional data, it cannot be excluded that the association with IADL disability might be also a consequence of opioid use.

The association between depressive symptoms and opioid use among home care clients with cognitive impairment was not explained by the severity of pain, pain-related diseases or disabilities. This raises doubts whether behavioural or verbal signs of depressive symptoms and pain are confused for each other by nurses and physicians. Depressive symptoms are strongly and reciprocally associated with both pain [31] and dementia [32]. As improved care of depression has been noted to decrease the severity of pain among older adults [33], it would be interesting to see if the same could be achieved in older adults with cognitive impairment—or if improved management of pain would decrease depressive symptoms in this patient group.

Opioid use was associated with many pain-related diseases—arthritis, previous fractures and pressure ulcers—and with ADLs only among clients without cognitive impairment. In addition, the association between opioid use and cancer within previous 5 years was not seen in the subpopulation of clients with cognitive impairment. It remained unclear in this setting why disabilities in ADLs were associated with opioid use only among clients without cognitive impairment. It might be that clients with cognitive impairment have often the ADL disability related to cognitive impairment itself, and among clients without cognitive impairment the ADL disability could more often be related to pain-related conditions instead, and for this reason be associated with opioid use. According to previous studies, pain related to arthritis [34] and cancer [35] may be misidentified and undertreated among patients with Alzheimer’s disease. Problems in recognising the pain of patients with cognitive impairment, however, does not explain why opioid use was associated with cardiac diseases and Parkinson’s disease among home care clients with cognitive impairment. More studies are needed to understand the characteristics and physical symptoms that affect the treatment of pain in people with cognitive impairment. Also, it should be examined more accurately in the future how pain itself affects the associations with home care clients’ opioid use.

Constipation was the only symptom associated with daily opioid use, whereas other symptoms in the RAI-HC, which could represent adverse effects of opioids based on previous knowledge [36], were not associated with opioid use in the adjusted analyses. This finding does not suggest that home care clients using opioids do not have these adverse effects. Clients experiencing dizziness used opioids less frequently after adjustments. This might be due to cautiousness in prescribing opioids to clients already suffering from symptoms which could be intensified by opioid use. The same kind of phenomenon, yet not statistically significant, was observed with difficulties in urination and delusions. Altogether, the frequency of some symptoms was quite low, questioning the ability of the RAI-HC to capture potential adverse drug effects. Especially constipation was reported only by 3% of opioid users, while in a systematic review about every third opioid user aged 65 years or older has reported constipation [12], and there is no tolerance effect for opioid-induced constipation [36]. Laxative use was not known, so constipation was either well treated or heavily unreported in this home care client population. Presumably, clients with several or serious adverse effects or events have stopped opioid use, so the population in the present study is selected.

The present study has certain strengths and limitations. Opioid users were identified based on the list of all medication included in the RAI-HC, and opioid use was confirmed by checking electronic medical records. Therefore, it is unlikely that some opioid users in this study were actually non-users, and vice versa. In the catchment area, circa 85% of home care clients were assessed with the RAI-HC during 2014, so the representativeness of the population of interest was quite good. Despite the often challenging context, the RAI-HC assessments have been considered reliable [16], and missing values were very rare in the present study. Although there may be miscoding or inaccurate evaluations at the level of the individual client, this should not systematically bias the present observations.

Degenerative spinal disorders and some conditions associated with neuropathic pain could not be taken into account in the analyses because they are not systematically recorded in the RAI-HC. Additionally, a part of diagnosed osteoarthritis (included in the category of arthritis) seemed to be missing from the RAI-HC compared to the data in the electronic medical records [1]. This missing part, however, probably comprises mainly clients with no or minor symptoms, because electronic medical records include all radiologically verified osteoarthritis independent of whether it causes any symptoms or disabilities. Frequencies of other diagnoses in the RAI-HC matched very well with the electronic medical records. The association of daily opioid use with somnolence or fatigue could not be analysed, as the RAI-HC does not include these symptoms. It is also acknowledged that the Pain scale is a relatively rough way to measure pain. Finally, it should be noted that following from the cross-sectional nature of the data, causes and consequences cannot be separated in the associations reported.

## Conclusions

The pain of home care clients with cognitive impairment may not be treated optimally. There might be incautious prescribing practices and prolonged opioid use without a sufficient evaluation of current pain among clients with osteoporosis, cancer within previous 5 years and disabilities in IADL. Most symptoms identified in the RAI-HC that could represent the adverse effects of opioids were not associated with the clients’ opioid use. This might reflect cautious prescribing practices and the highly selected population of mainly long-term users who tolerate opioids, or else the incapability of the RAI-HC to capture these symptoms.

## Data Availability

The Resident Assessment Instrument data that support the findings of this study were used under licence for the current study, and so the data are not publicly available.

## Appendix

Associations of daily opioid use with sociodemographic and clinical characteristics among aged home care clients with and without cognitive impairment

**Table:**

Characteristics	Cognitive impairment (*n* = 1284)			No cognitive impairment (*n* = 1275)		
	Unadjusted	Multivariable model 1	Multivariable model 2	Unadjusted	Multivariable model 1	Multivariable model 2
	OR (95% CI)	Adjusted OR (95% CI)	Adjusted OR (95% CI)	OR (95% CI)	Adjusted OR (95% CI)	Adjusted OR (95% CI)
Age (year)	1.03 (1.00–1.06)	1.01 (0.98–1.04)	1.01 (0.97–1.04)	0.99 (0.97–1.01)	0.98 (0.95–1.00)	0.98 (0.95–1.00)
Gender, female	1.35 (0.85–2.14)	1.00 (0.60–1.67)	0.95 (0.55–1.64)	1.45 (0.97–2.18)	1.37 (0.87–2.18)	1.03 (0.63–1.68)
Disability in ADLs (≥ 1)	0.99 (0.66–1.51)	0.67 (0.40–1.11)	0.65 (0.39–1.11)	**2.71** (1.89–3.90)	**1.91** (1.24–2.94)	**1.73** (1.10–2.74)
Performance in IADLs						
0–7	1	1	1	1	1	1
8–14	1.70 (0.88–3.30)	1.93 (0.94–3.95)	1.88 (0.90–3.93)	**1.84** (1.29–2.62)	**1.51** (1.03–2.21)	**1.54** (1.04–2.30)
15–21	**1.94** (1.02–3.71)	**2.21** (1.04–4.73)	**2.51** (1.15–5.48)	**2.51** (1.57–4.02)	1.35 (0.77–2.37)	1.48 (0.81–2.70)
Unstable health state (CHESS ≥ 3)	**1.65** (1.01–2.70)	1.05 (0.61–1.80)	0.80 (0.46–1.41)	**1.90** (1.08–3.35)	1.30 (0.70–2.42)	0.87 (0.46–1.66)
Depressive symptoms (DRS ≥ 3)	**2.96** (1.95–4.48)	**2.84** (1.81–4.44)	**1.70** (1.06–2.75)	**1.79** (1.11–2.87)	1.55 (0.93–2.60)	0.93 (0.54–1.62)
Chronic conditions						
Arthritis	1.19 (0.58–2.43)	0.94 (0.44–2.00)	0.81 (0.37–1.78)	**1.82** (1.15–2.88)	**2.08** (1.27–3.40)	**1.86** (1.10–3.13)
Cancer (in previous five years)	1.19 (0.58–2.43)	1.30 (0.61–2.77)	1.20 (0.54–2.63)	**1.70** (1.04–2.77)	**2.20** (1.29–3.75)	**2.03** (1.16–3.57)
Cardiac disease	**1.71** (1.14–2.58)	**1.69** (1.09–2.61)	1.56 (0.99–2.47)	0.88 (0.64–1.22)	0.88 (0.62–1.26)	0.87 (0.60–1.27)
Chronic renal failure	1.30 (0.65–2.58)	1.07 (0.52–2.20)	1.13 (0.53–2.40)	1.01 (0.59–1.73)	1.02 (0.57–1.83)	0.83 (0.45–1.52)
Diabetes	1.19 (0.77–1.85)	1.34 (0.85–2.14)	1.30 (0.80–2.10)	0.95 (0.67–1.34)	1.02 (0.70–1.49)	1.21 (0.81–1.80)
History of any fracture affecting the present health status	0.90 (0.38–2.11)	0.85 (0.35–2.06)	0.65 (0.26–1.61)	**2.33** (1.46–3.72)	**1.88** (1.14–3.11)	1.67 (0.98–2.84)
History of stroke	0.71 (0.32–1.57)	0.86 (0.37–1.95)	0.84 (0.36–1.99)	0.70 (0.37–1.34)	0.60 (0.30–1.20)	0.67 (0.32–1.41)
Osteoporosis	**2.51** (1.62–3.91)	**2.34** (1.44–3.79)	**1.84** (1.10–3.08)	**2.92** (2.08–4.11)	**2.81** (1.92–4.10)	**2.71** (1.81–4.06)
Parkinson’s disease	2.45 (0.99–6.05)	**3.09** (1.17–8.11)	**3.13** (1.11–8.83)	1.81 (0.81–4.04)	1.30 (0.53–3.22)	1.40 (0.54–3.66)
Peripheral arterial disease or venous insufficiency	0.94 (0.44–1.99)	0.86 (0.39–1.89)	0.68 (0.30–1.53)	1.14 (0.72–1.82)	0.97 (0.58–1.63)	0.87 (0.51–1.49)
Pressure ulcer	1.89 (0.78–4.59)	1.29 (0.47–3.54)	1.00 (0.34–2.93)	**2.43** (1.38–4.29)	**2.66** (1.42–4.96)	1.69 (0.86–3.31)
Pain scale						
No	1		1	1		1
Less than daily	6.38 (2.72–15.00)		5.69 (2.40–13.51)	2.55 (1.32–4.91)		2.38 (1.21–4.66)
Daily, mild to moderate	11.83 (5.24–26.67)		10.49 (4.57–24.05)	5.79 (3.20–10.47)		4.90 (2.64–9.07)
Daily, severe	33.68 (14.30–79.33)		27.27 (11.10–67.04)	18.70 (10.03–34.87)		16.03 (8.22–31.27)

Multivariable model 1 adjusted for all factors mentioned in the table except the Pain scale, and multivariable model 2 adjusted for all factors mentioned in the table
